# A Novel Strategy of Combined Pulsed Electro-Oxidation and Electrolysis for Degradation of Sulfadiazine

**DOI:** 10.3390/molecules28083620

**Published:** 2023-04-21

**Authors:** Dong Ma, Bo Zhang, Xiaomin Hu

**Affiliations:** Department of Environmental Engineering, School of Resource & Civil Engineering, Northeastern University, Shenyang 110819, China; 1510476@stu.neu.edu.cn (D.M.); 2010420@stu.neu.edu.cn (B.Z.)

**Keywords:** sulfadiazine, electro-activated, peroxymonosulfate, pulse current

## Abstract

A combination of the peroxymonosulfate (PMS) electro-activation process and the electro-oxidation process driven by a pulsed electric field (PEF) was used to degrade sulfadiazine (SND) wastewater. Mass transfer is the limiting step of electrochemical processes. The PEF could enhance mass transfer efficiency by reducing the polarization effect and increasing the instantaneous limiting current compared with the constant electric field (CEF), which could benefit the electro-generation of active radicals. The degradation rate of SND after 2 h was 73.08%. The experiments investigated the effects of operating parameters of pulsed power supply, PMS dosage, pH value and electrode inter distance on the degradation rate of SND. The predicted response value of single-factor performance experiments was obtained as 72.26% after 2 h, which was basically consistent with the experimental value. According to the quenching experiments and EPR tests, both SO_4_^•−^ and •OH were present in the electrochemical processes. The generation of active species were significantly greater in the PEF system than that in the CEF system. Moreover, four kinds of intermediate products were detected during the degradation by LC-MS. This paper presents a new aspect for electrochemical degradation of sulfonamide antibiotics.

## 1. Introduction

Sulfadiazine (SND), as a sulfonamide antibiotic, is widely used in clinical practice all over the world [1,2,3]. However, the degradation rate of SND in conventional municipal wastewater treatment systems is very low due to its strong biological resistance; thus, it continues to be discharged into natural water bodies. As reported, SND residual in surface water and ground water has caused a series of health issues and environmental problems [4,5]. Therefore, effective technology for SND degradation in water is a hot topic of recent research.

Advanced oxidation Processes (AOPs) based on sulfate radical (SO_4_^•−^) are currently considered to be one of the most effective methods for the treatment of organic pollutants in water [6,7,8,9,10,11]. SO_4_^•−^ itself has a lone pair of electrons, has a high oxidation capacity and can mineralize most of organic pollutants. Compared with •OH (E^0^(•OH/H_2_O) = 1.9–2.7 V), SO_4_^•−^ (E^0^(SO_4_^•−^/SO_4_^2−^) = 2.5–3.1 V) has a higher redox potential in a wider pH range and has a higher reaction rate with refractory organic pollutants in water [10,12,13]. PMS activated by Transition metal ions [14], heating [15], ultraviolet light [16,17], activated carbon [9,11,18] and electric field [19,20,21] was commonly used to produce •OH and SO_4_^•−^ due to its own asymmetric structure. However, these activation methods need to add large numbers of oxidants, and some of the catalysts are difficult to recover, which can easily cause secondary pollution. Among all these methods, electro-activated PMS oxidation system was easy to control and little secondary contaminants. Furthermore, organic matters could be oxidized to smaller molecules through electrochemical oxidation [20,22,23,24].

(1) $${\mathrm{HSO}}_{5}^{-}+e^{-}\to {\mathrm{OH}}^{-}+{\mathrm{SO}}_{4}^{•-}\mathrm{or}\;{}^{•}OH+{\mathrm{SO}}_{4}^{2-}$$

(2) $$H_{2}O\to {}^{•}OH+H^{+}+e^{-}$$

Selvendiran Periyasamy et al. [25] used graphite as the anode to explore the degradation of acetaminophen by electrochemical oxidation. The optimal process conditions were as follows: pH 4, acetaminophen concentration 20 mg/L, constant current density 5.1 mA/cm^2^, electrolyte Na_2_SO_4_ concentration 0.1 M. After 240 min of reaction, the acetaminophen removal rate was 90%. However, mass transfer steps are the limiting steps of electrochemical processes because electron transfer steps between electrode and electrolyte might be significantly strengthened by improving the electrode potential [20,22]. How to improve the mass transfer efficiency is one of the key topics of electrochemical research. As for some reports [26,27], the limiting value of the instantaneous pulse current for electrodeposition can be considerably higher than that of direct constant current plating attributed to the modulation of mass transport. It could be concluded that the usage of pulsed pattern instead of constant pattern might improve the mass transfer in interface between electrode and electrolyte.

In this study, PMS as an oxidant was activated by pulsed electric field (PEF/PMS), and graphite was used as the anode and cathode in an open cell electrolysis system. The subject of this paper is to: (1) investigate the effects of operating factors on SND degradation in the PEF/PMS system; (2) compare the degradation efficiencies of different systems; (3) inspect contrast of reactive species generation in PEF/PMS and CEF/PMS; (4) speculate the degradation pathway of SND in PEF/PMS.

## 2. Results and Discussion

### 2.1. Effects of Operating Parameters on SND Degradation Rates

The effects of pulse supply voltage, pulse frequency, pulse supply duty cycle, PMS concentration, initial pH value and electrode spacing on SND degradation in the PEF/PMS system were investigated (Text S3).

**Figure 1:**
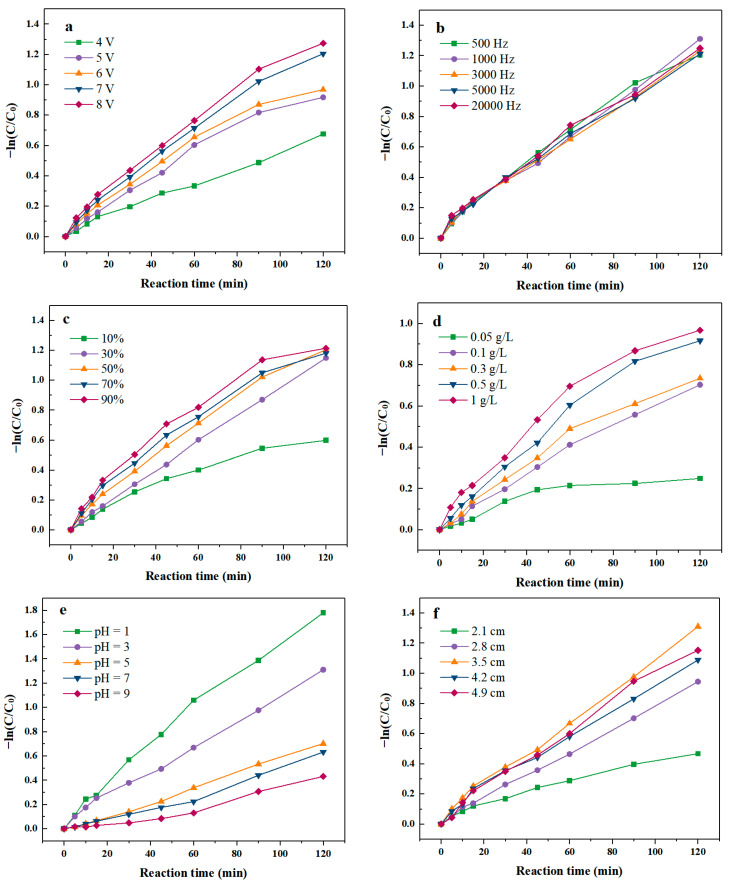
Effect of operation parameters on degradation rate of SND. (**a**) Voltage of PEF; (**b**) Frequency of PEF; (**c**) Duty cycle of PEF; (**d**) Initial PMS concentration; (**e**) Initial pH value; (**f**) Electrode inter distance.

Applied voltage is an important parameter of electrochemical reaction because change the potential of electrode may change the activation energy of electrochemical reaction [28,29]. According to Figure 1a, the degradation rate of SND increased from 48% to 73% as the applied voltage increased from 4 V to 8 V. With the increase in voltage in a certain range, the SND degradation rate gradually improved, indicating that voltage has a significant effect on the degradation of SND by pulse activated PMS (Table S1). When the voltage increased from 4 V to 5 V, the degradation rates of SND increased by 12%. However, the growing momentum waned when the voltage increased from 7 V to 8 V, and the degradation rates of SND increased by only 2%. Magnifying the pulse voltage can hasten the generation of electron, enhance the transfer of electrons and promote the electro-generation of SO_4_^•−^ and •OH by PMS activation [30,31,32]. Furthermore, with the increase in voltage, the oxygen generation at the anode was intensified, which was conducive to the removal of intermediates on the electrode surface, and the promotion of degradation of pollutants in the system. As shown in Table S2, the degradation rate constant of the pulse voltage 7 V and 8 V was similar. In addition, high voltage caused water electrolysis, resulting in instability of the degradation system; the most suitable pulse supply voltage was chosen to be 7 V.

Pulse frequency is one of the important factors that makes PEF different from CEF [27,33]. The frequency of the pulse is the reciprocal of the pulse period, and its extent can reflect the number of “on–off” pulse wave per second [34]. Figure 1b shows that when the frequency was 500 Hz, the degradation rate of SND was 42%. Increasing the frequency in the range of 1000 Hz–5000 Hz, the SND degradation rates were maintain at ~70%, which is about 30% higher than that of 500 Hz. However, the degradation rate of SND decreased to 53% as the frequency increased to 20,000 Hz. When the frequency was 3000 Hz, SND degradation rate reached the maximum value of 73%. The pulse “on–off” mode might develop a double diffusion layer that reduced the influence of polarization effect then increased the degradation efficiency of SND. In addition, the period “on–off” reduced the electrostatic repulsion between PMS and cathode, make PMS more capable to be activated at cathode surface. When the frequency is 3000 Hz, the fitting degree of degradation rate of SND is the largest, so the optimal frequency of the PEF was 3000 Hz.

The duty cycle of PEF refers to the percentage of the “on” time in one working cycle of a pulse, which is an important parameter of the PEF [33]. To explore the effect of different duty cycle on SND degradation by PEF/PMS, the duty cycle of PEF was set at 10%, 30%, 50%, 70% and 90%. As shown in Figure 1c, with the growth of duty cycle of PEF, the degradation rate of SND increased initially and then became steady. The lowest degradation rate of SND was 43% as the duty cycle was 10%. When the duty cycle was low, the on time in a pulse was comparatively low, the reaction time was limited and a lower amount of SO_4_^•−^ and •OH was produced in the system. When the duty cycle increased to 30%, the SND degradation rate improved to 68%. The ions adsorbed on the plate surface returned to the bulk solution temporarily in the process of SND degradation by PEF/PMS during the power off, reducing the difference in concentration between the bulk electrolyte and electrode/electrolyte interface. Thus, the concentration polarization effect was weakened and the reaction rate was promoted continuously. When the duty cycle was 50%, the degradation rate of SND was 70% after 2 h of reaction. After that, the degradation rates of SND were stable at about 70% with the rise of duty cycle. When the duty cycle was too high, the “on” time was comparatively long in a pulse, then the PEF system became close to CEF system which concentration polarization effect was easier to occur at the interface increasing the mass transfer resistance, hindered the degradation of SND [26,33,35]. The degradation rate constant *k* of SND was shown in Table S3, when the duty cycle of PEF is lower than 50%, the value of duty cycle has a significant impact on the degradation performance of SND by PEF/PMS. However, when the duty cycle was higher than 50%, there was no significant difference in SND degradation rate constants when the duty cycle continuous to increase. Then the optimal duty cycle of the PEF was selected as 50%.

As depicted in Figure 1d, the degradation efficiency of SND increased significantly when the PMS dosage increased from 0.05 g/L to 1 g/L in the system. When the concentration of PMS was 0.05 g/L, SND degradation efficiency was only 22%. With the increase in the initial PMS concentration to 0.1 g/L, the degradation rate of SND was significantly increased to 49%, indicating that a high concentration of PMS was more likely to be activated to produce •OH and SO_4_^•−^ enhancing SND degradation. However, when the dosage of PMS further increased, the ratio of SND degradation rates vs. PMS dosage dropped. This is because too much SO_4_^•−^ was generated instantaneously, and a high concentration of SO_4_^•−^ will self-quench, as depicted in Equations (3) and (4) [30,36,37]. Although more SO_4_^•−^ was produced, it does not participate in the SND degradation reaction. In addition, the remaining SO_4_^•−^ also reacts with HSO_5_^−^ in the system to form SO_5_^•−^ which is less active than •OH and SO_4_^•−^ [38]_._

SO_4_^−^ + SO_4_^−^ → S_2_O_8_^2−^ (3)

SO_4_^−^ + S_2_O_8_^2−^ → SO_4_^2−^ + S_2_O_8_^−^ (4)

HSO_5_^−^ + SO_4_^−^ → SO_4_^2−^ + SO_5_^−^ + H^+^ (5)

According to Figure 1e, acidic conditions might be of benefit to SND degradation by PEF/PMS. When the initial pH of the solution was chosen as 1, the degree of SND degradation was 82% after 2 h of reaction, then the degradation rates of SND decreased with the increasing of pH value. This was because acidic conditions were favorable to the formation of •OH and SO_4_^•−^, as well as in favor of their stable existence, thus enhancing the degradation performance of SND. Compared with •OH, the aromatic π electron was more likely to be attacked by electrophiles SO_4_^•−^ [39]. Moreover, the lifetimes of •OH and SO_4_^•−^ are relatively longer [11,15,40,41]. With the increase in pH, OH^−^ in the solution reacts with SO_4_^•−^, leading to the decrease in •OH and SO_4_^•−^ in the system. Another important reason that the acidic condition was more suitable for SND electrochemical degradation is that SND was more likely to protonate under acidic conditions, which makes SND more vulnerable to SO_4_^•−^ attack. Ionization equilibria constant pKa of SND also contributes effects on the changed rule. It has been widely reported that pKa of SND is around 6.5, which implies SND in solution are mainly in species carrying positive charge when pH is 3.5.

SO_4_^−^ + OH^–^ → SO_4_^2−^ + OH (6)

The electrode inter spacing could affect the current density and stirring effect in the SND degradation by PEF/PMS [29,42]. As depicted in Figure 1f, when the electrode spacing increased from 2.1 cm to 3.5 cm, the SND degradation rate augmented from 35% to 72%. However, the SND degradation rate decreased to 65% as the spacing increased to 4.2 cm. The stirring efficiency changed with the distance of plate. When the electrode spacing was relatively close, the stirring was insufficient, the thin liquid layer on the electrode surface could not be updated in time, resulting in the decreasing of degradation rate of SND (Table S4). However, too-wide spacing was not conducive to the degradation of antibiotics, because the internal resistance of the system was relatively large, reducing the electrical efficiency of the system. When the electrode spacing was 3.5 cm, the degradation rate of SND was maximum, so the optimal spacing was 3.5 cm in the experiment.

**Figure 2:**
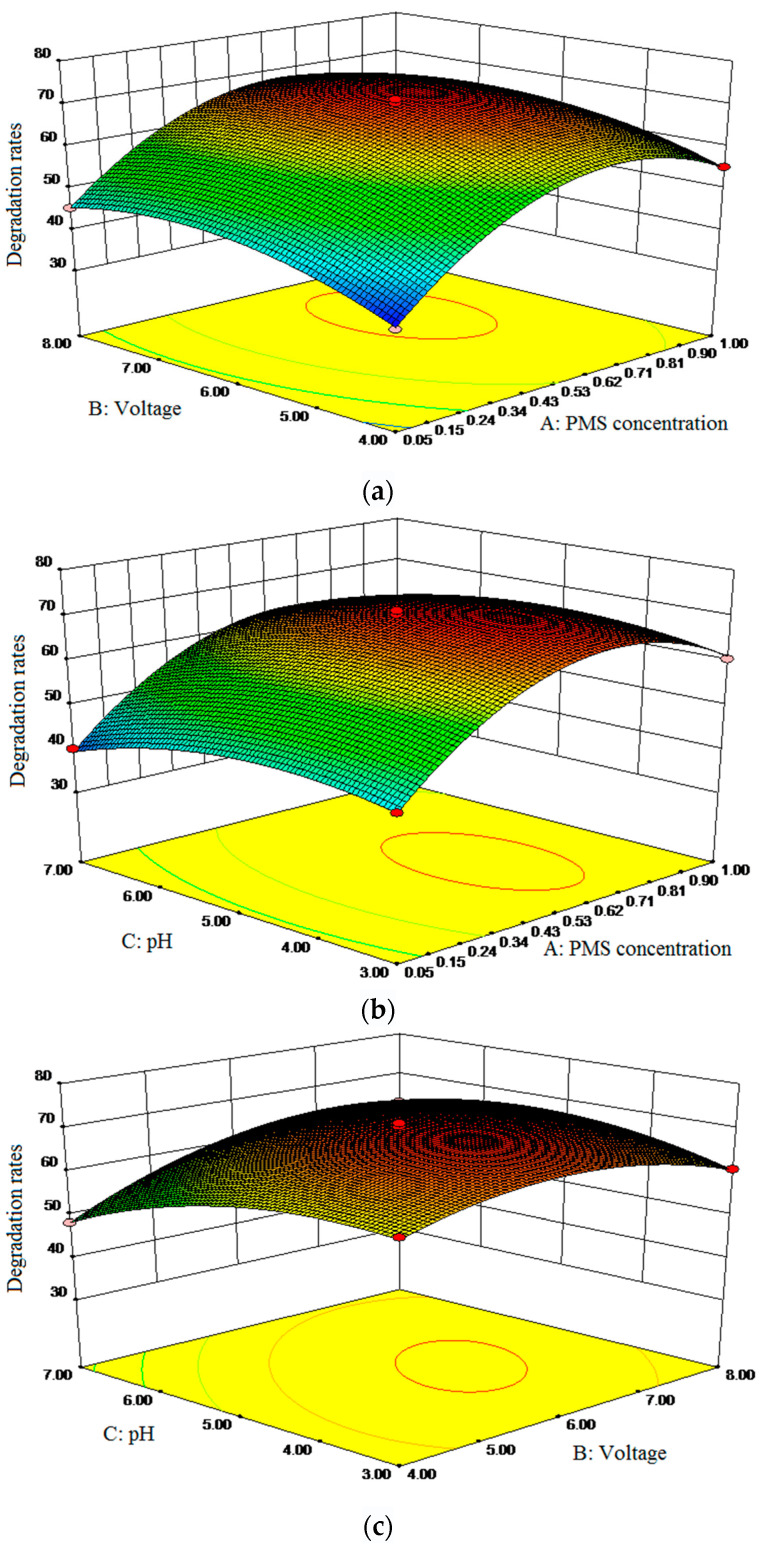
Interaction effect of single parameters on the degradation rate of CIP. (**a**) PMS concentration and voltage; (**b**) PMS concentration and pH; (**c**) pH and voltage.

Design Expert was used to calculate the optimal process parameters and predict influence value (Figure 2). Response surface methodology (RSM) analysis (Text S1) [17,43] was conducted to identify the influence of single factors. As the exploration of the influence of single parameters on SND degradation by PEF/PMS, applied voltage, PMS dosage and pH value were selected as the factors and SND degradation rate was used as the response value in response surface test design (Tables S5 and S6). Three levels were set for each factor.

The prediction results were verified through experiments, and the results were shown in Table 1. the optimal process parameters were as follows: pH value was 4.73, voltage was 6.44 V, PMS concentration was 0.63 g/L. The predicted response value: SND degradation rate was 71.72%. Under the optimal process parameters, the predicted response value was verified. After three repeated tests, the average SND degradation rate was 72.26%, which was basically consistent with the predicted value, indicating that the extraction process parameters were reliable and had practical application value.

In PEF/PMS system, SND degradation was mainly affected by free radical oxidation, which was easily affected by inorganic ions in a water environment. Therefore, the effects of NO_3_^−^ and Cl^−^ on SND degradation were investigated. As depicted in Figures S2–S4, both Cl^−^ and NO_3_^−^ can inhibit SND degradation by PEF/PMS. They would compete with SND in the reaction system for active free radicals, resulting in a decrease in SND degradation rate. The inhibitory effect of Cl^−^ on SND degradation was greater than NO_3_^−^ [39].

Cl^−^ + SO_4_^•−^ → SO_4_^2−^ + Cl• (7)

Cl^−^ + •OH → OH^−^ + Cl• (8)

### 2.2. Compare of Different Systems

**Figure 3:**
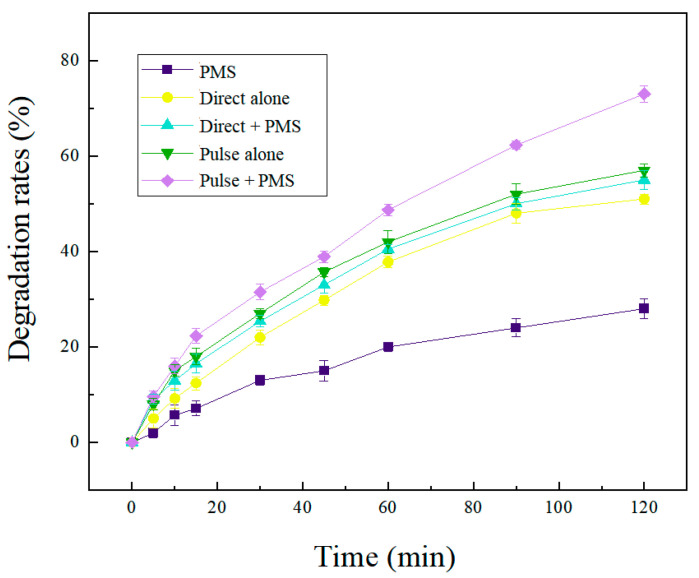
Effect of different reaction systems on the degradation effect of SND.

For further investigation of the effect of different systems on the SND degradation, the PEF/PMS, CEF/PMS, PEF, CEF and PMS system were analyzed (Table S7). It can be seen from Figure 3 that the degradation rate of SND varied greatly with different reaction systems. The degradation rate of SND degraded with PMS alone was the lowest, only 18% after 2 h reaction. It has been reported that PMS can be activated at room temperature (22 C), but the oxidation effect was weak so the degradation of SND was not substantial. In the CEF system, the SND degradation rate was 50%. In the CEF treatment system, there were two main reasons for SND oxidation: (i) direct oxidation by anode; SND was oxidized by direct electron transfer reaction on the anode surface. (ii) Mediate oxidation by active species generated by electrolysis; •OH generated on the graphite anode surface by water electrolysis which has an indirect oxidation effect on SND. H_2_O_2_ and •O^2−^ produced by the cathodic reduction as shown in Equations (9) and (10) [44] also have an indirect oxidation effect on SND.

O_2_ + 2H^+^ + 2e^−^ → H_2_O_2_ (9)

O_2_ + e^−^ → •O^2−^ (10)

The degradation efficiency of SND was significantly improved with addition of PMS in the CEF/PMS treatment system compared with the CEF system. HSO_5_^−^ was electro-activated to produce SO_4_^•−^ and •OH, which could degrade SND into smaller molecular organic matters [9].

HSO_5_^−^ + e^−^ → SO_4_^•−^ + •OH (11)

In the electro-activated PMS system, both O_2_ and HSO_5_^−^ would approached the graphite electrode surface to compete for electrons and convert to H_2_O_2_ and SO_4_^•−^, respectively. However, since SO_4_^•−^ has stronger oxidation capacity and was easier to obtain electrons than H_2_O_2_, the reaction system was more inclined to promote electron transfer and activate PMS to produce SO_4_^•−^. Moreover, H_2_O_2_ was not detected in electrolyte. As reported by Liu et al. [22] no accumulation of H_2_O_2_ was found in the electrochemically activated PMS system.

In comparison with CEF/PMS, the degradation rate of SND by PEF/PMS was 20% higher, and the degradation rate of SND by PEF/PMS system was higher than that of CEF/PMS system at every sampling point. Moreover, the degradation rate of SND after 240 min reached 90%. In the CEF, the concentration distribution on the electrode surface is roughly linear. At this time, the electrode surface is in a steady state condition, and the reactant consumption and diffusion mass transfer are in a dynamic balance. The control step of the electrode process is the mass transfer process at the electrode/electrolyte interface, and concentration polarization occurs on the electrode surface. Under the pulse condition, the electrode surface concentration gradient is very steep, the reactant on the electrode surface is rapidly consumed and the electrode process is in a transient state. At the early stage of the reaction, the electrode process is not controlled by mass transfer, and the instantaneous current density is higher and the electrode reaction speed is faster. When the pulse “on” condition ended, the pulse “off” condition provide diffusion mass transfer near the electrode surface (Table S8). At this time, the concentration gradient of the electrode surface slowed down, completing the reactant supplement and reducing the influence of the electrode surface concentration polarization [26,27,33,34,45].

By comparing the five systems, it could be concluded that there might be four main oxidation mechanism of PEF/PMS system: (i) SND directly oxidized on the anode surface by electron transfer; (ii) PMS in the reaction system has oxidation property, which could degraded SND; (iii) The indirect anode oxidation of SND by the SO_4_^•−^ and •OH produced by the anodic electrolysis of water and sulfate ions; (iv) degradation of SND by electro-activated PMS generated •OH and SO_4_^•−^.

### 2.3. Identification of Radicals in PEF/PMS System

Electro-activated PMS can produce not only SO_4_^•−^, but also •OH, and both radicals can oxidize antibiotics at very high rate constants (k_(•OH SND)_ = 3.6 × 10^9^; k_(SO_4_^•−^ SND)_ = 4.16 × 10^10^). In order to investigate the existence of SO_4_^•−^ and •OH in the reaction system, the free radical quenching experiment was carried out. Tert-butanol (TBA) and methanol (MeOH) were selected as inhibitors of •OH and SO_4_^•−^, respectively, to explore their effects on SND (Table S10) degradation by electrically activated PMS system. The reaction rate between quenching agents and radicals were shown in Table S9 [17,46,47].

**Figure 4:**
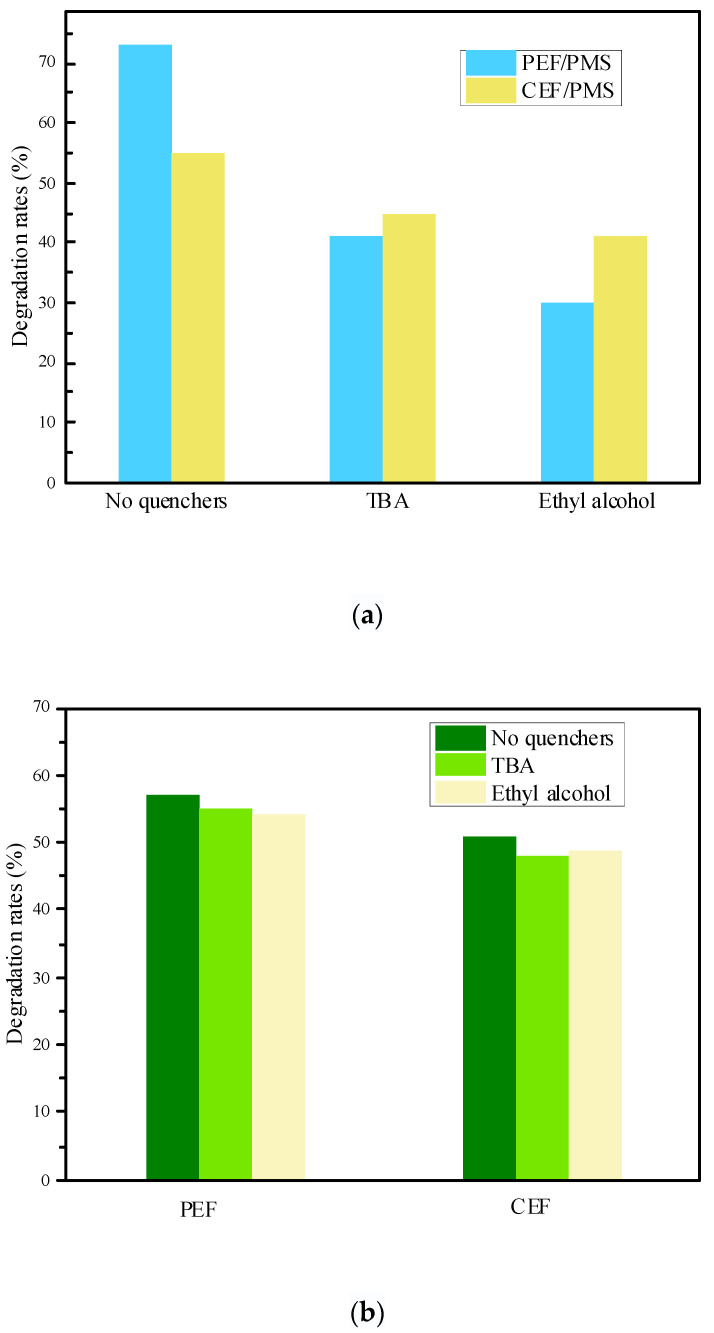
Effect of free radical inhibitors on SND degradation in the system. (**a**) Effect of free radical inhibitors on SND degradation in different electro-activated PMS systems; (**b**) Effect of free radical inhibitors on SND degradation in the different electric field system. Concentrations of quenchers were 0.5 mol/L.

As shown in Figure 4a, the SND degradation with different quenchers in the PEF/PMS system were 72%, 40% and 30%, respectively, indicating that SND in the PEF/PMS system was partially degraded by free radical oxidation. The degradation rate of SND reduced by 32% as the addition of TBA, which was mainly caused by the quenching of •OH in the system, indicating the generation of •OH in the system. The degradation rate of SND decreased by 43% when MeOH was added, indicating that both SO_4_^•−^ and •OH were generated in the system. In contrast, the difference between TBA inhibition and MeOH inhibition was 10%, indicating that the amount of SO_4_^•−^ in the system was less than that of •OH. On the other hand, •OH plays a certain role on the formation of SO_4_^•−^. In addition, degradation rates of SND under different inhibitor conditions in the CEF/PMS system were 54%, 44% and 40%, respectively. By comparing with the PEF/PMS system, it can be seen that the quenching effect of inhibitors on SND degradation in the CEF/PMS system is far less than that in the PEF/PMS system under the same conditions, which further proved that the PEF is more likely to activate PMS to produce more •OH and SO_4_^•−^.

As shown in Figure 4b, SND degradation under different quenching conditions in the PEF system was 56%, 54% and 53%, respectively. While in CEF system, the degradation rates were 50%, 47% and 48%, respectively. In the two electric field systems, the decreasing trend of SND degradation rate is not obvious, which indicates that the amount of •OH and SO_4_^•−^ produced by electrochemistry in the system is very low. The fractions of quenching by quenchers in CEF/PMS and PEF/PMS were estimated in Table S11. By comparing the CEF/PMS system, it could be seen that the inhibition effect of free radical inhibitor on SND degradation in the CEF/PMS system was much greater than that in the CEF system under the same conditions, indicating that more active radicals were produced in the CEF/PMS system, which further proved that in the CEF/PMS system, SND was mainly oxidized and degraded by free radicals produced by electro-activated PMS.

In order to confirm that active species play a more important role in PEF/PMS, EPR test of electrochemical system was performed in situ (Text S2). As shown in Figure S4, •OH and SO_4_^•−^ signal peaks were clearly found in the CEF system, indicating that both •OH and SO_4_^•−^ were generated by electrolysis of water and sulfate ions on the electrode surface existed in the system, indicating that the degradation of SND in the CEF electrochemical oxidation system was mainly affected by •OH and SO_4_^•−^. Typical four-fold and six-fold signals were also found in the CEF/PMS system, indicating that •OH and SO_4_^•−^ are both generated in the CEF/PMS system. The signal intensity peaks of •OH and SO_4_^•−^ were higher than that of CEF because •OH and SO_4_^•−^ could be generated after PMS activation, and more •OH and SO_4_^•−^ were present in the system, not only generated from activated PMS, but also from direct electrochemical oxidation (Figure S5). In addition, both •OH and SO_4_^•−^ were also detected in PMS system. By comparing the two electro-activated PMS systems, it was obvious that the signal intensity of •OH and SO_4_^•−^ was stronger in PEF/PMS system, indicating that more •OH and SO_4_^•−^ were produced in PEF/PMS system. Therefore, it can be concluded that the sequence of •OH and SO_4_^•−^ electro-generation capacity of the three systems under the same condition was: PEF/PMS > CEF/PMS > CEF. This conclusion was consistent with the previous quenching experimental results.

### 2.4. Degradation Pathway of SND in PEF/PMS System

In order to explore the degradation pathway of SND during the degradation of SND by PEF/PMS, the reaction intermediates were detected by HPLC-MS/MS.

**Figure 5:**
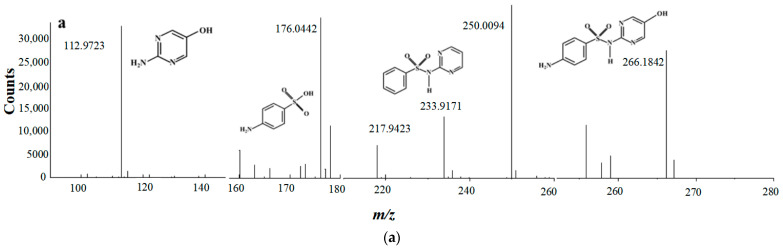

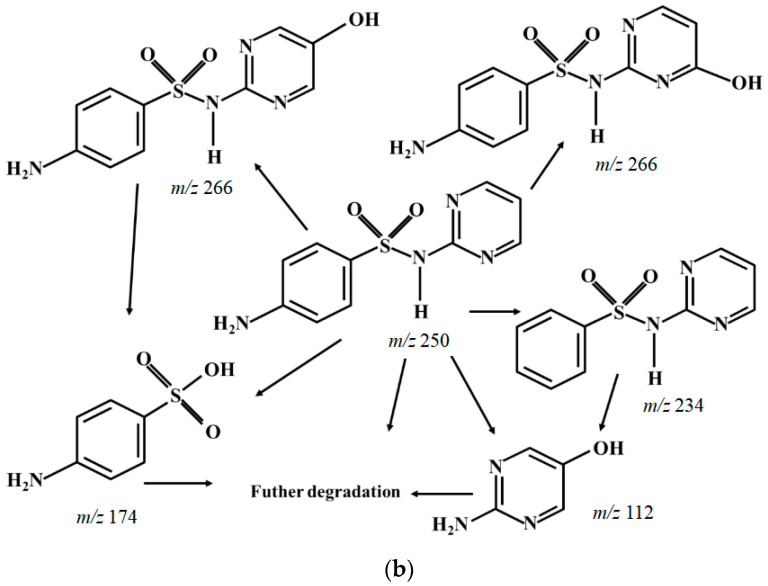
The main transformation pathways of SND. (**a**) MS spectra of SND degradation byproduct; (**b**) Hypothesis of degradation pathways of SND.

As shown in Figure 5a, four typical intermediates are detected, which are *m*/*z* 112, *m*/*z* 234, *m*/*z* 174 and *m*/*z* 266, respectively. It has been widely reported that pKa of sulfadiazine is around 6.5 [48], which implies sulfadiazine in solution is mainly in a species carrying positive charge when pH is 3.5. As shown in Figure 5b, there might be three main pathways for SND degradation. Firstly, SND (*m*/*z* 250) attacked by SO_4_^•−^ and/or •OH, produces *m*/*z* 266 by substitution reaction [47], then *m*/*z* 174 is produced by loss of pyrimidine group [47,49], and then further degradation. Secondly, SND may also undergo deamination to produce *m*/*z* 234. Fragment *m*/*z* 218 of *m*/*z* 234 by loss of –NH_2_, was detected, and further generated *m*/*z* 174 and *m*/*z* 112 [50]. Thirdly, active species SO_4_^•−^ and/or •OH oxidized sulfamine structure of SND and produced *m*/*z* 174.

## 3. Materials and Methods

### 3.1. Chemicals

All solutions were prepared using analytical grade reagents and Milli-Q water. All chemicals were used as received without any additional purification and were of reagent grade purity. SND was purchased from Macklin (Shanghai, China). Other reagents all purchased from Sinopharm Chemical Reagent (Shanghai, China). 5,5-dimethyl-1-pyrroline-*N*-oxide (DMPO) are purchased from Sigma–Aldrich (St. Louis, MO, USA).

### 3.2. Experimental Setup

All the experiments were carried out in a 300 mL glass reactor (Figure S1). Simulated wastewater with SND concentration of 0.1 g/L were prepared. Concentrations of PMS (2KHSO_5_∙KHSO_4_∙K_2_SO_4_, aq 10 g/L) were 0.05 g/L, 0.1 g/L, 0.3 g/L, 0.5 g/L and 1.0 g/L. Applied voltages of the pulse power supply were 4 V, 5 V, 6 V, 7 V and 8 V. Duty cycles of pulse power supply were 10%, 30%, 50%, 70% and 90%. Frequencies of pulse power supply were 500 Hz, 1000 Hz, 3000 Hz, 5000 Hz and 20,000 Hz. pH values were adjusted by NaOH and H_2_SO_4_ to 1, 3, 5, 7 and 9. Electrode inter distance were 2.1 cm, 2.8 cm, 3.5 cm, 4.2 cm and 4.9 cm. Different concentrations of Cl^−^ (NaCl, aq 1 mol/L) and NO_3_^−^ (NaNO_3_, aq 1 mol/L) were 0 mol/L, 0.01 mol/L, 0.05 mol/L and 0.1 mol/L.

Graphite was used as the anode and cathode. The pulse power supply and constant power supply were used as power device. The magnetic stirrer was set at 600 R/min and the reaction time was set at 120 min. Samples were taken after every 0 min, 5 min, 10 min, 15 min, 30 min, 45 min, 60 min, 90 min and 120 min in the experiment. In order to study the reaction kinetics, the first order reaction kinetics was fitted to the degradation time of SND. Degradation rate *R* and reaction rate constant *k* were calculated by Equations (12) and (13), respectively. *C*_0_ is the initial mass concentration of SND, mg/L; *C* is the mass concentration of SND after *t* reaction time, mg/L [51,52].

(12) $${R=(C}_{0}{-C)/C}_{0}\;\times \;100\%$$

(13) $$k={-\mathrm{dln}(C/C}_{0})/\mathrm{dt}\;$$

### 3.3. Analysis Methods and Instruments

The conductivity and pH were measured with a Metrohm 644 conductometer (Crison Instruments, Barcelona, Spain) and a Crison 2000 pH-meter (Crison Instruments, Barcelona, Spain), respectively. Constant current electrolysis was performed with an Amel 2049 (Amel, Italy) potentiostat galvanostat and pulse current electrolysis was performed with an SOYI–2010M (Dalian Zhongxing Electronic Technology Co., Ltd., Dalian, China) high frequency pulse power supply. The potential difference between the anode and cathode (Ecell) was provided by a Demestres 601BR digital multimeter (Demeter (Shanghai) Environmental Protection Equipment Co., Ltd., Shanghai, China.). The decomposition of antibiotics was assessed by high performance liquid chromatography-mass spectrometry/mass spectrometry (HPLC-MS/MS) [53]. A Shimadzu LC-20AD system (Shimadzu, Kyoto, Japan) was equipped with a 2.0 mm × 100 mm C18 column (Shimadzu, Kyoto, Japan) [54,55]. A Bruker A300 spectrometer (Billerica, MA, USA) was used for electron paramagnetic resonance (EPR) analysis with DMPO and TMP as spin-trapping agents for SO_4_^•−^ and •OH [6,56].

## 4. Conclusions

In this study, the simulated wastewater was prepared with typical antibiotic sulfadiazine. The graphite electrode was driven by pulse power supply, and the PMS was used as oxidant to evaluate the treatment efficiency of PEF/PMS for degradation of antibiotics in water. The results showed that the PEF was more suitable for the electrocatalytic oxidation process than the traditional CEF. The voltage, frequency and duty cycle of the pulse field were adjusted. Factors such as PMS dosage, initial pH value and electrode distance were investigated. The degradation effect of SND was evaluated as the target pollutant. The optimal working condition was selected as voltage 7.0 V, frequency 3000 Hz, duty cycle 50%, [PMS] = 0.5 g/L, pH = 3.5, electrode distance = 3.5 cm. The quenching experiments showed that more reactive oxidative species were produced in PEF/PMS. HPLC-MS/MS was used to analyze the substances in the solution. Four main intermediates were detected. Loss of pyrimidine group, desulphidation and deamination might be the most possible pathway of SND degradation. This study provided a theoretical and technical foundation for the application of PEF/PMS in the treatment of refractory organic wastewater.

## Figures and Tables

**Table 1:** The predicted and experimental values under optimized process parameters.

pH	Voltage (V)	PMS Concentration (g/L)	Predicted Value (%)	Experimental Value (%)
4.73	6.44	0.63	71.72	72.26

## Data Availability

The data that support the findings of this study are available from the corresponding author upon reasonable request.

## Supplementary Materials

The following supporting information can be downloaded at: https://www.mdpi.com/article/10.3390/molecules28083620/s1.

## Sample Availability

Samples of the compounds are available from Dong Ma.
