# Supplementary material for: A Novel Strategy of Combined Pulsed Electro-Oxidation and Electrolysis for Degradation of Sulfadiazine
Source: Molecules. 2023 Apr 21;28(8):3620. doi: 10.3390/molecules28083620 (PMC10142080; doi:10.3390/molecules28083620)
Supplement: Supplementary file 1 [file molecules-28-03620-s001.zip › molecules-2310201-supplementary.pdf]

SUPPORTING INFORMATION

A Novel Strategy of Combined Pulsed Electro-oxidation and Electrolysis for Degradation of Sulfadiazine

Dong Ma[#]*, Bo Zhang*[#]*, Xiaomin Hu**

Department of Environmental Engineering, School of Resource & Civil Engineering,

Northeastern University, Shenyang 110819, China

Phone & Fax: +86-24-83680401
Email: hxmin_jj@163.com

[#]These authors contributed equally to this study.

***Corresponding Author:**

Xiaomin Hu, Northeastern University, Email: hxmin_jj@163.com, Phone & Fax: +86-24-83680401

Table of Contents

Text S1. Response surface methodology (RSM) analysis

Text S2. EPR instrumental parameters.

Text S3. Analytical methods of byproducts.

Table S1. First order kinetics of SND degradation with different PMS concentration

Table S2. First order kinetics of SND degradation with different frequency

Table S3. First order kinetics of SND degradation with different pH

Table S4. First order kinetics of SND degradation with different electrode inter distance

Table S5. First order kinetics of SND degradation with different impulse voltage

Table S6. First order kinetics of SND degradation with different duty cycle

Table S7. First-order kinetics of SND degradation with different reaction systems

Table S8. Variance analysis for the established regression model

Table S9. Reaction rate of radical inhibitors with hydroxyl and sulfate radicals

Table S10. Chemical and physical character of SND.

Table S11 Contribution rate of active species oxidized substance degradation by PMS activated by different electric fields.

Figure S1. Diagram of experimental device.

Figure S2. Effect of NO_3^- concentration on SND degradation.

Figure S3. Effect of Cl^- concentration on SND degradation.

Figure S4. ESR spectra at three reaction systems.

Figure S5. Effect of operation parameters on degradation rate of SND. (a) Initial PMS concentration; (b) Voltage of PEF; (c) Frequency of PEF; (d) Duty cycle of PEF; (e) Initial pH value; (f) Electrode inter distanceRefreneces.

Test S1. Response surface methodology (RSM) analysis

According to the Box-Benhnken design prinSNDle, the response surface test results were fitted to the second-order model data, and the regression equation was obtained as follows:

SND degradation rate =70.07＋8.20A＋3.41B－2.64C－0.81AB＋0.26AC＋4.04BC－14.28A^2－6.31B^2－5.21C^2

Test S2. EPR instrumental parameters

The center field of the spectrometer was 3897 G, resonance frequency (empty) of 9.81 GHz. The signal of free radicals that were trapped by 5,5-dimethyl-pyrroline-oxide (DMPO). The sample solution and 50 μM DMPO solution were mixed quickly at a ratio of 2:1 (v/v) to get adducts. Operating parameters: Center field, 3385 G; Static field, 3285 G; sweep width, 200 G; Modulation frequency, 100 kHz; Modulation amplitude, 2.00 G; Microwave bridge frequency, 9.52 GHz; Power, 2.00 mW. Four equidistance and highly intense peaks with intensity ratio of approximately 1:2:2:1 and six low intensity peaks whose ratio was approximately 1:1:1:1:1:1 as signed to DMPO−OH and DMPO–OSO_3H, respectively. The hyperfine splitting constants for the modeled spectra were as follows: aH = aN = 14.9 G for DMPO−OH; aN = 12.2 G, aH = 10.28 G, 2.58 G and 0.1 G for DMPO–OSO_3H.

Test S3. Analytical methods of byproducts.

For characterization of SND degradation by HPLC-MC/MS (Agilent Technologies, USA), the Column - C18 (2.1 × 100 mm, 1.7 μm) was used. The mass spectrometric analysis was conducted using positive/ negative electrospray ionization with a mass scan range of m/z 50-1000. The capillary voltage, cone voltage, desolvation temperature, and source temperature were set at 2000 V, 40 V, 450 °C, and 115 °C, respectively.

10 μL of the samples was injected into column with the mobile phases of 10mM ammonium acetate/methanol (10/90, v/v). The flow rate was set at 0.2 mL min^{-1} and the column temperature maintained at 30 C, operated with electro spray ionization (ESI) in negative mode.

Tables

Table S1 First order kinetics of SND degradation with different PMS concentration

PMS（g/L）	k/min^{-1}	R^2
0.05	2.2×10^{-3}	0.8414
0.1	6.02×10^{-3}	0.9933
0.3	6.35×10^{-3}	0.9761
0.5	8.01×10^{-3}	0.9775
1	8.25×10^{-3}	0.9589

Table S2 First order kinetics of SND degradation with different frequency

Frequency（Hz）	k/min^{-1}	R^2
500	4.74×10^{-3}	0.9744
1000	1×10^{-2}	0.9881
3000	1.04×10^{-2}	0.9958
5000	9.73×10^{-3}	0.9941
20000	6.45×10^{-3}	0.9782

Table S3 First order kinetics of SND degradation with different pH

pH	k/min^{-1}	R^2
1	$1.45×10^{-2}$	0.9906
3	$1.04×10^{-2}$	0.9958
5	$5.9×10^{-3}$	0.9965
7	$5.13×10^{-3}$	0.9801
9	$3.54×10^{-3}$	0.9497

Table S4 First order kinetics of SND degradation with different electrode inter distance

Electrode inter distance（cm）	k/min^{-1}	R^2
2.1	3.79×10^{-3}	0.9772
2.8	7.67×10^{-3}	0.9984
3.5	1.04×10^{-2}	0.9958
4.2	8.71×10^{-3}	0.9929
4.9	9.49×10^{-3}	0.9942

Table S5 First order kinetics of SND degradation with different voltage

Voltage（V）	k/min^{-1}	R^2
4	5.36×10^{-3}	0.9929
5	8.01×10^{-3}	0.9775
6	8.3×10^{-3}	0.9704
7	1×10^{-2}	0.9877
8	1.06×10^{-2}	0.9848

Table S6 First order kinetics of SND degradation with different duty cycle

Duty cycle（%）	k/min^{-1}	R^2
10	5.14×10^{-3}	0.9536
30	9.52×10^{-3}	0.9994
50	1×10^{-2}	0.9877
70	9.85×10^{-3}	0.9693
90	1.02×10^{-2}	0.9523

Table S7 First-order kinetics of SND degradation with different reaction systems

Reaction systems	k/min^{-1}	R^2
PMS alone	2.72×10^{-3}	0.9624
Direct alone	6.17×10^{-3}	0.9724
Direct+PMS	6.6×10^{-3}	0.9763
Pulse alone	6.97×10^{-3}	0.9725
Pulse+PMS	1.04×10^{-2}	0.9958

Table S8 Variance analysis for the established regression model

Sources of variation	Quadratic sum	Degree of freedom	Mean square	F	P	Clinical significance
Model	1995.20	9	221.69	381.89	＜0.0001	**
A	537.59	1	537.59	926.08	＜0.0001	**
B	92.75	1	92.75	159.78	＜0.0001	**
C	55.76	1	55.76	96.05	＜0.0001	**
AB	2.59	1	2.59	4.47	0.0725	
AC	0.26	1	0.26	0.45	0.5247	
BC	65.45	1	65.45	112.74	＜0.0001	**
A^2	859.08	1	859.08	1479.89	＜0.0001	**
B^2	167.59	1	167.59	288.70	＜0.0001	**
C^2	114.47	1	114.47	197.18	＜0.0001	**
Residual error	4.06	7	0.58			
Lack of fit	1.90	3	0.63	1.17	0.4255	
Pure error	2.17	4	0.54			
Total variation	1999.26	16				
R^2					0.9980	
R^2_{Adj}					0.9954	
R^2_{Pred}					0.9831	

**P≤0.01 means factors have a significant impact on the response value.

Table S9 Reaction rate of radical inhibitors with hydroxyl and sulfate radicals

Radical inhibitors	Rate constants/（mol·L^{-1}·s^{-1}）	
	•OH	SO_4^{-}·
SND	8.78×10^{9}	4.16×10^{10}
MeOH	9.7×10^{8}	3.2×10^{6}
Ethanol	（1.2～2.8）×10^{9}	（1.6～7.7）×10^{7}
TBA	（3.8～7.6）×10^{9}	（4～9.1）×10^{5}
Nitrobenzene	（3～3.9）×10^{9}	1.2×10^{5}

Table S10 Chemical and physical character of SND.

Characters	Value
Molecule structure	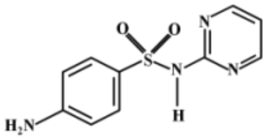<br>C10H10N4O2S
Molar mass	250.28
pK_{a1}	2.49
pK_{a2}	6.48

Table S11 Contribution rate of active species oxidized substance degradation by PMS activated by different electric fields

	$SO_4^{\bullet -}$	•OH
PEF/PMS	12	30
CEF/PMS	4	10

Figures

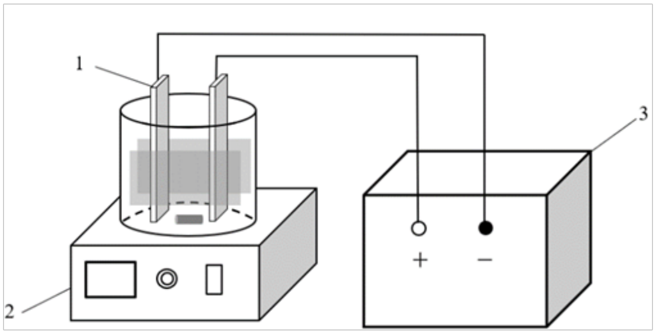

1. Graphite electrode; 2. Magnetic stirring controller; 3. Power supply.

Figure S1. Diagram of experimental device.

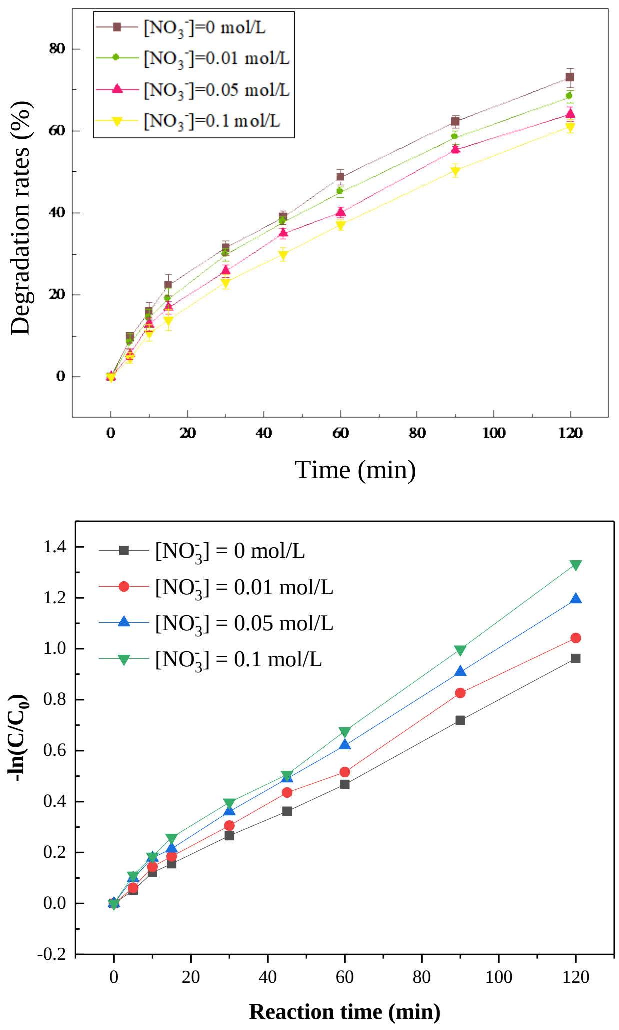

Figure. S2 Effect of NO_3^- concentration on SND degradation

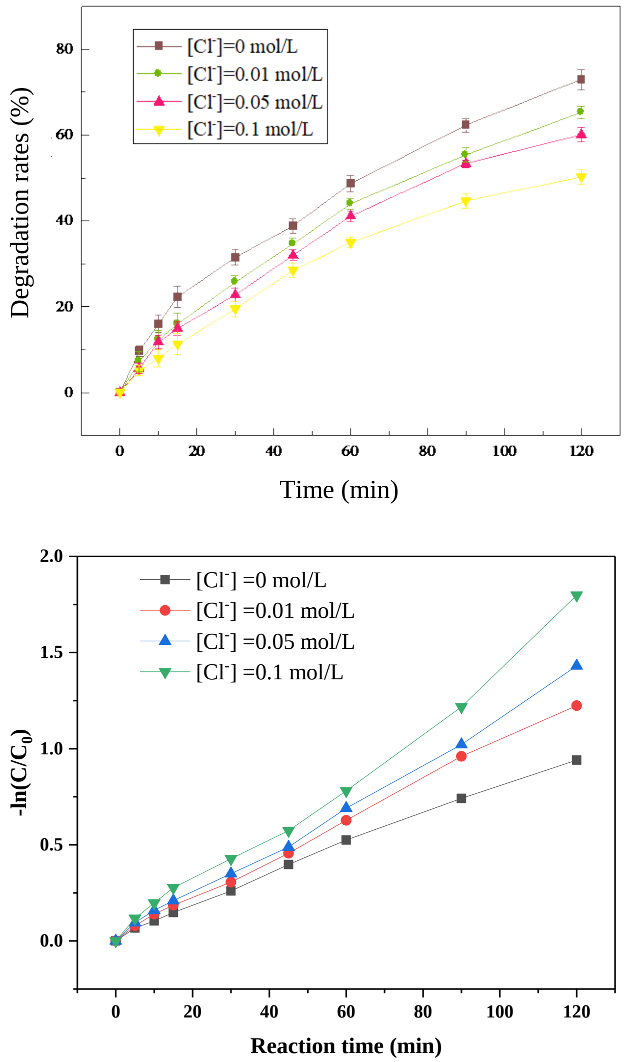

Figure. S3 Effect of Cl^- concentration on SND degradation

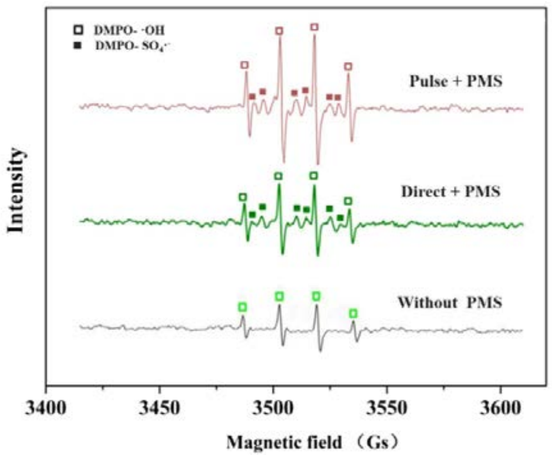

Figure S4. ESR spectra at three reaction systems

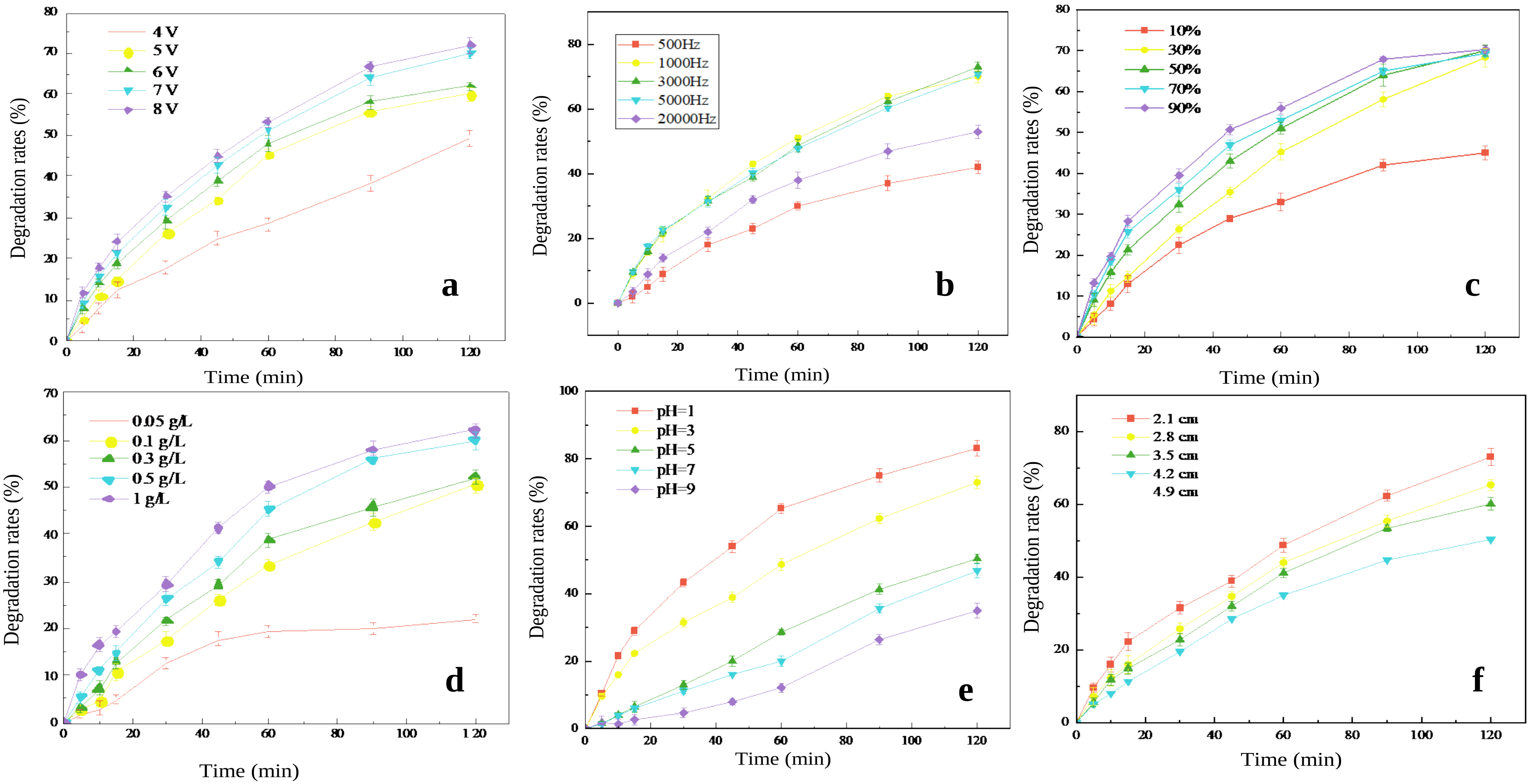

Figure S5. Effect of operation parameters on degradation rate of SND. (a) Initial PMS concentration; (b) Voltage of PEF; (c) Frequency of PEF; (d) Duty cycle of PEF; (e) Initial pH value; (f) Electrode inter distance
